# Copy Number Variation in Subjects with Major Depressive Disorder Who Attempted Suicide

**DOI:** 10.1371/journal.pone.0046315

**Published:** 2012-09-27

**Authors:** Roy H. Perlis, Douglas Ruderfer, Steven P. Hamilton, Carl Ernst

**Affiliations:** 1 Department of Psychiatry, McGill University, Montreal, Quebec, Canada; 2 Center for Human Genetic Research, Massachusetts General Hospital, Harvard Medical School, Boston, Massachusetts, United States of America; 3 Department of Psychiatry, University of California San Francisco, San Francisco, California, United States of America; University of Iowa Hospitals & Clinics, United States of America

## Abstract

**Background:**

Suicide is one of the top ten leading causes of death in North America and represents a major public health burden, partcularly for people with Major Depressive disorder (MD). Many studies have suggested that suicidal behavior runs in families, however, identification of genomic loci that drive this efffect remain to be identified.

**Methodology/Principal Findings:**

Using subjects collected as part of STAR*D, we genotyped 189 subjects with MD with history of a suicide attempt and 1073 subjects with Major Depressive disorder that had never attempted suicide. Copy Number Variants (CNVs) were called in Birdsuite and analyzed in PLINK. We found a set of CNVs present in the suicide attempter group that were not present in in the non-attempter group including in SNTG2 and MACROD2 – two brain expressed genes previously linked to psychopathology; however, these results failed to reach genome-wide signifigance.

**Conclusions:**

These data suggest potential CNVs to be investigated further in relation to suicide attempts in MD using large sample sizes.

## Introduction

Major depressive disorder (MD) is a debilitating illness affecting approximately 5–15% of people in the United States [1], resulting in economic burdens such as lost work days [2] and decreased life expectancy in affected individuals [3]. Symptoms include lack or gain of sleep, weight gain/loss, feelings of hopelessness, depressed mood, and lack of motivation, amongst others, as defined both by the International Classification of Disease and the Diagnostic and Statistical Manual. Phenotypic heterogeneity has long been seen as a major confounding factor in genetic studies of MD [4], and suicide attempts represent a documentable outcome of some people with MD, possibly representing a sub-group of individuals with MD.

MD and suicide run in families, suggesting that they might partially be explained by genetics [5], [6]. Still, while most studies of the genetics of MD and suicide show a high heritability estimate [7]–[10], it is unclear which genes may be driving the effect. Heritability studies led to candidate gene approaches in search of genetic variants that may be passed from one affected generation to another. Genes involved in biological pathways of antidepressant action (candidates such as 5HTR2A, MAO-A, and 5-HTT), were screened for variation that might associate with disease. This approach largely proved challenging [11], with low reproducibility across studies. To avoid searching for variation associated with disease in *a priori* genes, genome-wide approaches have been applied where hundreds of thousands of variants can be screened at once [12]–[14]. Another approach to identify genes of interest related to disease is to perform genome-wide searches for copy gains and losses [4], [15], instead of investigations of single nucleotide polymorphisms. While these copy number variants (CNVs) are not intrinsically more pathogenic than a single nucleotide change, they are large and have the potential to increase or decrease gene product at each CNV that intersects a gene, or alter the genomic environment with potentially far-reaching *cis* or *trans* effects.

The purpose of the current study was to examine and identify CNVs in people with MD who had attempted suicide and determine if these differ from MD cases that never attempted suicide. We reasoned that suicide attempters with MD might represent a genetically different group from people with MD who never attempted suicide. We used whole-genome SNP microarrays to call CNVs >100 Kb and then assessed CNV frequency differences between people with MD who either had or had not attempted suicide, as well as in a non-psychiatric control group.

## Materials and Methods

All protocols and sample collections were approved by the IRB of the Massachusetts General Hospital and all data were analysed anonymously.

The STAR*D cohort [16] has been extensively used in many genetic studies and has been thoroughly documented [17]. Lifetime history of suicide attempts was assessed at the initial study visit by the study clinician and suicidal behavior was not exclusionary for the initial STAR*D patient recruitment, provided the patient did not require hospitalization [18].

Genotyping for the STAR*D cohort utilized the Affymetrix GeneChip Human Mapping 500 K Array Set and the Affymetrix Human SNP Array 5.0 [19]. Genotypes for samples run on the Affymetrix 500 K Array (n = 969) were called using the BRLMM algorithm, and those analyzed on Affymetrix Array 5.0 (n = 979) were called using the BRLMM-P algorithm. Additional QC was performed using PLINK [20], where individuals or SNPs were excluded with total call-rates <95%, SNPs with call rates <98%, individuals with minor allele frequencies <1%, or out of Hardy-Weinberg equilibrium (p<1×10−6). We imputed missing genotypes using MACH and retained SNPs with r2>0.8. Eleven subjects were excluded due to missing clinical data resulting in a final dataset of 1, 262.

CNVs were identified using Birdseye [21], which identifies CNVs by integrating intensity data from neighboring probes using a hidden Markov model (HMM) on a per- individual basis. Performance is dependent on a number of factors including SNP and copy number probe density, mean intra-individual probe variance and CNV frequency. For each CNV a LOD score was generated that describes the likelihood of the CNV relative to no CNV over the given interval. All CNV analysis were performed in PLINK and only those CNVs present in less than 10% of the total sample were used for analysis.

Secondary controls for the current study came from the Database of Genomic Variants (http://projects.tcag.ca/variation/), a database of over 100, 000 CNVs from over 40 studies. While these studies do not explicitly screen for mood disorders, only controls from these studies are in the database.

## Results

The STAR*D dataset comprised 1,262 individuals (483 males), where 189 cases attempted suicide while 1,073 did not attempt suicide. In all analyses, we clustered data using PLINK to account for population stratification which in this dataset comprised three groups (Caucasian, African-american, and Hispanic). In all cases, we assessed only those CNVs that were greater than 100 Kb. While admittedly a conservative number, CNV call accuracy increases proportionally to predicted CNV size. Utilization of this CNV size for analysis is consistent with previous work [22].

We first asked whether there was a difference in copy number burden between people with MD who attempted suicide compared to those that had never attempted suicide. We found no significant difference in CNV burden defined as CNV size, total Kb spanned, or proportion of CNVs/person, when assessing deletion or duplication CNVs (Tables 1 and 2).

**Table 1:** Burden of deletion CNVs >100 Kb in subjects with major depression that did (MD_SA) or did not attempt suicide (MD_NO SA).

TEST	GRP	MD_SA	MD_NO SA	p
CNVs	ALL	111	613	-
RATE	ALL	0.5873	0.5713	0.84
PROP	ALL	0.3598	0.4231	0.09
TOTKB	ALL	405.7	326.9	0.10
AVGKB	ALL	243.5	235.5	0.77
CNVs	afr	23	125	
CNVs	cau	74	414	
CNVs	his	14	74	
RATE	afr	0.5227	0.625	
RATE	cau	0.6549	0.564	
RATE	his	0.4375	0.5324	
PROP	afr	0.4091	0.485	
PROP	cau	0.3628	0.4114	
PROP	his	0.2812	0.3957	
KBTOT	afr	379	338.4	
KBTOT	cau	443.6	321.6	
KBTOT	his	285.9	335.7	
KBAVG	afr	294.8	249.2	
KBAVG	cau	233.6	229.8	
KBAVG	his	185.9	242.5	

Abbreviations: CNVs: Number of segments; PROP: Proportion of sample with one or more segment; TOTKB: Total kb length spanned by all segments per individual; AVGKB: Average segment size. His: Hispanic; afr: African-American; cau: Caucasian.

**Table 2:** Burden of duplication CNVs >100 Kb in people that did (MD_SA) or did not attempt suicide (MD_NO SA).

TEST	GRP	MD_SA	MD_NO SA	p
N	ALL	154	827	-
RATE	ALL	0.814	0.7707	0.570
PROP	ALL	0.534	0.5433	0.874
TOTKB	ALL	514.8	471.5	0.335
AVGKB	ALL	330.8	335.6	0.875
N	afr	39	168	
N	cau	92	548	
N	his	23	111	
RATE	afr	0.8864	0.84	
RATE	cau	0.8142	0.7466	
RATE	his	0.7188	0.7986	
PROP	afr	0.5455	0.57	
PROP	cau	0.5398	0.5395	
PROP	his	0.5	0.5252	
KBTOT	afr	518.2	497.9	
KBTOT	cau	511	469.3	
KBTOT	his	524.5	442.1	
KBAVG	afr	322.1	349.8	
KBAVG	cau	330.6	343.4	
KBAVG	his	344.7	270.9	

Abbreviations: CNVs: Number of segments; PROP: Proportion of sample with one or more segment; TOTKB: Total kb length spanned per indivudal; AVGKB: Average segment size. His: Hispanic; afr: African-American; cau: Caucasian.

Next, we asked whether there was an increased probability of a CNV intersecting a given gene between suicide attempters and non-attempters. To do this we assessed the number of CNVs in both groups that intersected any gene. Point tests were performed for each gene and two-sided p-values were generated comparing probability estimates permuting over the whole genome (Table 3). Presented in Table 3, we show all CNVs that differed between MD_SA (suicide attempt) and MD_NO SA (no suicide attempt) at single point p-value<0.1. We observed no genome-wide significant hits; however, we note that more CNVs that intersected genes were present in the MD_SA group than in the MD_NO SA group, suggesting that MD_SA may be a more severe phenotype than people with MD_NO SA. All CNVs intersecting genes were duplications, except for MACROD2, a gene previously linked to Autism [23]. We also analyzed these data using genome-wide correction, by CNV type (statistics generated separately for deletions and duplications – Table 4).

**Table 3:** Genomic location of all CNVs >100 Kb that disrupt genes.

Chr	Gene	p-value	p-value (genome)	Start (hg18)	End	MD_SA	MD_NO SA
2	LOC391343	0.022	0.818	892822	896011	2	0
2	SNTG2	0.022	0.818	936554	1350391	2	0
9	KANK1	0.049	0.976	494702	736103	2	1
10	PPYR1	0.019	0.988	46503539	46508326	13	35
10	MTG1	0.056	0.976	135057610	135084164	2	1
10	SPRN	0.056	0.976	135084159	135088111	2	1
20	FLRT3/MACROD2	0.017	0.818	14252642	14266270	2	0
22	ZNF74	0.083	0.976	19078479	19092752	2	1
22	SCARF2	0.083	0.976	19108874	19122146	2	1

Only loci that show single point p-values less than 0.1 between attempters and non-attempters are shown. Abbreviations: hg18: UCSC human genome build 18 coordinates. MD_SA: Subjects with Major Depressive disorder who attempted suicide; MD_NO SA: Subjects with Major Depressive disorder who never attempted suicide.

**Table 4:** Genomic location of CNVs >100 Kb that intersect genes across all subjects, analyzed by CNV type.

CNV	Chr	Gene	p-value	p-value (genome)
DUP	2	LOC391343	0.032	0.785
DUP	2	SNTG2	0.032	0.785
DUP	9	KANK1	0.054	0.949
DUP	10	PPYR1	0.004	0.866
DUP	10	MTG1	0.057	0.949
DUP	10	SPRN	0.057	0.949
DEL	20	FLRT3/MACROD2	0.025	0.273

Only CNVs with single point p-values<0.1 are presented. DEL = deletion CNV; DUP = duplication CNV.

To determine if CNVs that intersected genes might be pathogenic, we screened the database of genomic variants to determine if any control subjects had CNVs that intersected any of these genes, matched for CNV type (i.e., deletion or duplication). All CNVs that intersect genes identified in the MD_SA population have been previously reported.

To determine if any regions of the genome had differences in CNV number, irrespective of whether they intersected genes, between MD_SA and MD_NO SA, we performed an identical analysis as with those CNVs that intersect genes; however, we found no significant differences in any genomic regions in CNV number (Table 5).

**Table 5:** CNVs from any region of the genome that show differences between MD_SA and MD_NO SA, with single point p-values<0.1 (Hg18 coordinates).

Chr	Start	Stop	p-value	p-value (genome)	MD_SA	MD_NO SA	CNV
2	780000	1140000	0.035	0.989	2	0	DUP
5	104380000	104480000	0.023	0.989	2	0	DEL
6	57560000	57700000	0.023	0.989	2	0	DUP
7	61740000	61860000	0.024	0.997	3	2	DUP
9	520000	900000	0.024	0.989	2	0	DUP
9	30160000	30460000	0.036	0.989	2	0	DUP
10	47160000	47260000	0.012	0.997	13	35	DUP
20	13960000	16040000	0.014	0.989	2	0	DEL

## Discussion

We performed an analysis of copy number variation (CNV) in people with Major Depressive disorder (MD) who had previously attempted suicide and compared CNVs from this population to people with MD that had never made a suicide attempt. Our results suggest that no CNV distinguishes these two groups, and that if a particular CNV is associated with suicide attempts in MD, it would likely be a common CNV. That is, we did not find any CNVs not reported in the Database of Genomic Variants, suggesting that no copy changes influence suicide attempt status in the STAR*D sample.

Why didn't we detect a difference in CNV frequency or CNV burden between groups? While our study is large compared to most studies performed in psychiatric genetics, it was likely underpowered for the current analysis. The best CNV differences that we detected were 2∶0, which might suggest that a sample size 4–5 times larger might be able to detect an effect; however, given that all identified CNVs are present in greater than 1% of the general population, it is likely that merely increasing the sample size will also identify controls with similar CNVs. This suggests that CNVs do not contribute to suicide attempts in Major Depression, at least in the STAR*D sample, though it is possible that the presence of a common CNV in combination with a particular genetic background and environment increases risk. To detect an effect of a common CNV, sample sizes would need to be increased >20–30-fold over the current study design, at least following the analysis model employed in the here. Another explanation for the lack of significant results in this study is that despite using a well-annotated sample, the attempter and no attempter groups are still heterogeneous. Attempt status was determined by a single report about suicide history during enrollment for STAR*D – it may be that there is large variation in how subjects report suicide history. Future CNV studies in suicide may want to consider using comprehensive questionnaires about suicide history. For psychiatric genetics, this raises the interesting question of how homogenous a sample needs to be before attempting to find genetic variation associated with disease. For example, a study with a similar study design to the current one might use only young adults with major depression, separating case and control based on severity of suicide attempts, number of suicide attempts, and/or complete absence of suicide attempts. Statistics become challenging when these issues are addressed, but this in combination with very large sample sizes from ethnically and socio-economically homogenous groups may be what is required to identify genetic variation relevant to psychopathology. Finally, we note the technology for calling CNVs was lower resolution than could have been used. Specifically, we called CNVs from SNP genotyping arrays using very conservative calling criteria, which increased the false negative rate. For example, there may be smaller CNVs (CNVs less than 100 Kb were screened out in this study) that show a significant difference between attempters and non-attempters, or there may have been CNVs that did not meet signal intensity thresholds. In either case, the data analyzed was high quality but likely did not detect all CNVs present in the STAR*D sample. Utilization of Whole Genome Sequencing, array-Comparative Genomic Hybridization, or 1 M SNP arrays would have given better resolution for CNV detection.

The current study used conservative filtering criteria for CNV analysis and stringent QC measures for array analysis. We also took advantage of a well-documented sample set (STAR*D) where many other studies have also been performed, potentially allowing for further downstream analyses with other data generated from this sample.

## References

[1] Kessler RC, McGonagle KA, Zhao S, Nelson CB, Hughes M, et al (1994) Lifetime and 12-month prevalence of DSM-III-R psychiatric disorders in the United States. Results from the National Comorbidity Survey. Archives of general psychiatry 51: 8–19. 8279933 10.1001/archpsyc.1994.03950010008002

[2] Kessler RC, Frank RG (1997) The impact of psychiatric disorders on work loss days. Psychological medicine 27: 861–873. 9234464 10.1017/s0033291797004807

[3] Chang CK, Hayes RD, Perera G, Broadbent MT, Fernandes AC, et al (2011) Life expectancy at birth for people with serious mental illness and other major disorders from a secondary mental health care case register in London. PloS one 6: e19590. 21611123 10.1371/journal.pone.0019590 PMC3097201

[4] Pang AW, MacDonald JR, Pinto D, Wei J, Rafiq MA, et al (2010) Towards a comprehensive structural variation map of an individual human genome. Genome biology 11: R52. 20482838 10.1186/gb-2010-11-5-r52 PMC2898065

[5] Mondimore FM, Zandi PP, Mackinnon DF, McInnis MG, Miller EB, et al (2006) Familial aggregation of illness chronicity in recurrent, early-onset major depression pedigrees. The American journal of psychiatry 163: 1554–1560. 16946180 10.1176/ajp.2006.163.9.1554

[6] Brent DA, Oquendo M, Birmaher B, Greenhill L, Kolko D, et al (2002) Familial pathways to early-onset suicide attempt: risk for suicidal behavior in offspring of mood-disordered suicide attempters. Archives of general psychiatry 59: 801–807. 12215079 10.1001/archpsyc.59.9.801

[7] Weissman M (2009) Depression. Annals of epidemiology 19: 264–267. 19344866 10.1016/j.annepidem.2009.01.021 PMC2735878

[8] Sullivan PF, Neale MC, Kendler KS (2000) Genetic epidemiology of major depression: review and meta-analysis. The American journal of psychiatry 157: 1552–1562. 11007705 10.1176/appi.ajp.157.10.1552

[9] Brent DA, Bridge J, Johnson BA, Connolly J (1996) Suicidal behavior runs in families. A controlled family study of adolescent suicide victims. Archives of general psychiatry 53: 1145–1152. 8956681 10.1001/archpsyc.1996.01830120085015

[10] Dumais A, Lesage AD, Alda M, Rouleau G, Dumont M, et al (2005) Risk factors for suicide completion in major depression: a case-control study of impulsive and aggressive behaviors in men. The American journal of psychiatry 162: 2116–2124. 16263852 10.1176/appi.ajp.162.11.2116

[11] Munafo MR (2006) Candidate gene studies in the 21st century: meta-analysis, mediation, moderation. Genes, brain, and behavior 5 Suppl 1: 3–8. 10.1111/j.1601-183X.2006.00188.x 16417611

[12] Sullivan PF, de Geus EJ, Willemsen G, James MR, Smit JH, et al (2009) Genome-wide association for major depressive disorder: a possible role for the presynaptic protein piccolo. Molecular psychiatry 14: 359–375. 19065144 10.1038/mp.2008.125 PMC2717726

[13] Shi J, Potash JB, Knowles JA, Weissman MM, Coryell W, et al (2011) Genome-wide association study of recurrent early-onset major depressive disorder. Molecular psychiatry 16: 193–201. 20125088 10.1038/mp.2009.124 PMC6486400

[14] Muglia P, Tozzi F, Galwey NW, Francks C, Upmanyu R, et al (2010) Genome-wide association study of recurrent major depressive disorder in two European case-control cohorts. Molecular psychiatry 15: 589–601. 19107115 10.1038/mp.2008.131

[15] Lee C, Scherer SW (2010) The clinical context of copy number variation in the human genome. Expert reviews in molecular medicine 12: e8. 20211047 10.1017/S1462399410001390

[16] Rush AJ, Fava M, Wisniewski SR, Lavori PW, Trivedi MH, et al (2004) Sequenced treatment alternatives to relieve depression (STAR*D): rationale and design. Controlled clinical trials 25: 119–142. 15061154 10.1016/s0197-2456(03)00112-0

[17] Perlis RH, Huang J, Purcell S, Fava M, Rush AJ, et al (2010) Genome-wide association study of suicide attempts in mood disorder patients. The American journal of psychiatry 167: 1499–1507. 21041247 10.1176/appi.ajp.2010.10040541 PMC3795390

[18] Rush AJ, Trivedi MH, Ibrahim HM, Carmody TJ, Arnow B, et al (2003) The 16-Item Quick Inventory of Depressive Symptomatology (QIDS), clinician rating (QIDS-C), and self-report (QIDS-SR): a psychometric evaluation in patients with chronic major depression. Biological psychiatry 54: 573–583. 12946886 10.1016/s0006-3223(02)01866-8

[19] Garriock HA, Kraft JB, Shyn SI, Peters EJ, Yokoyama JS, et al (2010) A genomewide association study of citalopram response in major depressive disorder. Biological psychiatry 67: 133–138. 19846067 10.1016/j.biopsych.2009.08.029 PMC2794921

[20] Purcell S, Neale B, Todd-Brown K, Thomas L, Ferreira MA, et al (2007) PLINK: a tool set for whole-genome association and population-based linkage analyses. American journal of human genetics 81: 559–575. 17701901 10.1086/519795 PMC1950838

[21] Korn JM, Kuruvilla FG, McCarroll SA, Wysoker A, Nemesh J, et al (2008) Integrated genotype calling and association analysis of SNPs, common copy number polymorphisms and rare CNVs. Nat Genet 40: 1253–1260. 18776909 10.1038/ng.237 PMC2756534

[22] The International Schizophrenia Consortium (2008) Rare chromosomal deletions and duplications increase risk of schizophrenia. Nature 455: 237–241. 18668038 10.1038/nature07239 PMC3912847

[23] Anney R, Klei L, Pinto D, Regan R, Conroy J, et al (2010) A genome-wide scan for common alleles affecting risk for autism. Human molecular genetics 19: 4072–4082. 20663923 10.1093/hmg/ddq307 PMC2947401

